# The Story of the Insane from Year to Year

**Published:** 1894-02-17

**Authors:** 


### THE STORY OF THE INSANE FROM YEAR TO YEAR.

(Sixth Series.)

### WONFORD HOSPITAL FOR THE INSANE, EXETER.

A slight decrease in the numbers has to be noted, the year closing with 120 patients in the asylum. There were 25 admissions (including 3 re-admissions), 13 recoveries, and 5 deaths. When dealing with such small numbers the percentages for any single year do not mean much, but we find the recoveries stand at 52 per cent. of the admissions, and the deaths at 4 per cent. on the average number resident. Ten of the admissions were due to hereditary influence, and 2 to drink. There was no death caused by consumption. In former notices we have quoted Dr. Deas' remarks on mechanical restraint, and he now tells us that in one case of flesh-picking the treatment was so successful that not only was the habit abandoned, but the insanity cured. The average income per patient is £1 19s. 10d. a week. Of the total number of patients, 70 are paying less than they cost, and 19 of these paid less than 20s. a week, while 5 were maintained gratuitously.

---

### HANTS COUNTY ASYLUM, FAREHAM.

The numbers increased during the year from 952 to 1,004. There were 226 admissions (including 31 re-admissions), 61 recoveries, and 90 deaths. The percentage of recoveries stands at 26·9, and the deaths at 9·2. Intemperance in drink is the assigned cause of the insanity in 39 of the admissions, and hereditary influence in 62. Previous attacks are credited with 47. Of the deaths 31 were due to brain disease, 12 to consumption, and 2 to typhoid fever; but both the latter were epileptics, and in one of them it was difficult to say whether the fever or the epilepsy caused death. Dr. Worthington strongly urges the advisableness of building for idiot children, and the visitors are empowered to communicate with the Asylum Committees of Dorset and Wiltshire, as to the practicability of providing a joint asylum for the three counties. This is a good idea, and no doubt it would answer well if the Hants Committee are willing to put off building until the Greek Kalends. Dr. Worthington's alternative is to add a block to his own asylum; and there cannot be a doubt that this is the quickest, cheapest, and best course to pursue—experience at the Berry Wood Asylum has shown it to be not only practicable but profitable. Our congratulations are offered to Miss Alibone and George Moss, the former having received a pension of £65 a year, and the latter one of £56. The visitors say the "state and condition of the asylum are highly satisfactory." The weekly rate is 9s. 6½d.

---

### THE RAINHILL ASYLUM, LIVERPOOL.

The numbers rose from 1,761 to 1,804. The admissions were 465, the recoveries 177, and the deaths 188. Put into percentages, the recoveries would stand at 38·14 of the admissions, and the deaths at 10·45 of the average number resident. Intemperance in drink is credited with having caused the insanity in 135 cases, and hereditary influence in 106. More than one-half of the insanity of the year is thus shown to be due to preventable causes. Seventy-two of the deaths were due to brain diseases, and 39 to consumption.

Dr. Wiglesworth thinks that general paralysis of the insane is not only commoner than it was, but that it makes its appearance at an earlier age than it did formerly, and he tells us of a case dying of that terrible disease at the age of eighteen. Dr. Wiglesworth gives us no crumb of hope that it will decrease in the future. On the contrary, "we are not likely to see a diminution in the sufferers from this malady." The cost per head per week is a trifle over 9s.

